# Moliform Fibro-Cartilaginous Tumor, Attended with Enlargement and Obliteration of the Cavity of the Uterus

**Published:** 1844-11-16

**Authors:** J. Pancoast

**Affiliations:** Professor of Anatomy in Jefferson Medical College, &c.


### RECORD OF MEDICAL SCIENCE.

*Moliform Fibro-Cartilaginous Tumor, attended with Enlargement and Obliteration of the Cavity of the Uterus.* Successfully extracted by J. Pancoast, M. D., Professor of Anatomy in Jefferson Medical College, &c.

The patient in this case was a Mrs. L., a lady 37 years of age, residing in the upper part of the city, who had been for a considerable period under the professional care of Dr. John K. Knorr, of this place. From this gentleman I have received the following brief history, which is illustrative of the obscurity so commonly attendant on uterine tumors, previous to their making their appearance in the vagina.

"Thirteen years since, Mrs. L. aborted with a first child. A short time afterwards, feeling that all was not right with her, she consulted the physician then in attendance upon the family, who advised her to wear a pessary. A waxed cork pessary was accordingly introduced, which she wore for some weeks. After riding upon one occasion for some distance, on a rough road, she was seized with a severe pain in the lower part of the abdomen, accompanied with a discharge of blood from the vagina. This induced her physician to remove the instrument. Since that period, she has at different times consulted various physicians, all of whom agreed that she had merely prolapsus uteri. After she came under my charge, consultations were at my request held with two distinguished practitioners of this city, one of whom supposed there was a tumor of a carcinomatous character, covering a large portion of the posterior part of the uterus; the other, that there was a polypous or carcinomatous expansion of the posterior lip of the uterus. A remarkable feature in this case was the fact that she menstruated regularly till within a short time of the period at which you first saw her. During a few weeks prior to the operation, there was a discharge of pus from the tumor at intervals of several days; on one occasion as much as half a pint being discharged at a time. This discharge, it was hoped, might effect a reduction of the tumor, so as to protract the patient's life. No such good effect, however, seemed to follow; the tumor appeared to steadily increase in size, and the patient became altogether confined to her chamber. The success of the operation you performed has been perfect in this case, Mrs. L. now enjoying as good health as at any previous period of her life.

Very truly yours,

*To J. Pancoast, M. D.* J. K. Knorr."

When called to the case of Mrs. L., in consultation with Dr. Knorr, an uterine tumor could be felt on palpation over the hypogastrium, round and smooth, and about the size of the fœtal head. The vagina, on examination, was found filled with a globular mass at its upper part, from which a tail-like process protruded, with its free end between the labia. This end was about an inch and a half broad, and an inch thick; and from its exposure to the urine, and probably to the contact of foreign bodies, was almost black, diffusing a gangrenous odor. On tracing up the tumour with the finger, no line of demarcation could be distinguished between the morbid growth, and the rounded mass of the uterus itself, which could be felt at the top of the vagina. A small fissure, however, was detected on the side of the tail-like process which led into a smooth, narrow cavity a half an inch deep, the axis of which was in the direction of the centre of the mass above it. I was led to infer from the examination, and the great solidity of the uterine tumor, that this cavity had probably been the source of the purulent discharges, and that its present small size might be owing to a rapid growth of new substance from the whole inner face of the uterus, which had not only filled up the cavity of that organ, but shot out in the process discoverable from the vulva. I made an attempt to draw down the whole mass of the tumor by this process, so as to expose the point at which the expanded orifice of the uterus embraced the tumor, but the process was found too friable to admit the application of the requisite degree of force. The finger was then brought from the top of the vagina downwards, so as to search for the junction of the os tincæ with the process of the tumor, the process being pushed in the opposite direction with the other hand. By this means I was able to detect a little prominence, which proved to be a portion of the lip of the uterus, that had been overlapped and obscured by the tumor. The end of the forefinger was gradually worked under this prominence and carried round the tumor, so as to separate from the latter the inner face of the neck which adhered closely to it. The ends of the forefinger of each hand were then carried up on opposite sides of the tumor, to serve as points of support to each other, while the process of separation was continued as far up as the fingers would reach. The partially loosened mass was then grasped with the thumb and forefinger of one hand, so as to draw the tumor downward till the *os tincæ* came in view from the vulva, and the separation continued further with the forefinger of the other hand, which was carried up with its palmar face towards the uterine wall. When the tumor was detached from the uterine wall as far as the finger would reach, recourse was had to a small pair of the œsophagus forceps invented by Dr. Bond, of this city. This instrument was passed up closed, with the concave face towards the uterus, between the uterine wall and the adherent tumor; and the blades then opened with force, so as to continue the process of separation. By repeating this manœuvre on the several sides of the tumor, its loosening was effected up to the fundus of the uterus. The forceps were then passed up with their *concave face to the tumor*, and by using them partly in the manner described and partly as a lever, the whole tumor was detached from the uterus, and drawn out from the vagina in a mass, lacerated to some extent by the instrument. The tumor formed a bulk somewhat larger than the closed fist, and weighed three quarters of a pound. It seemed to have been attached equally to all parts of the internal face of the uterus, the line of attachment, as in cases of moles and adherent placentæ, being rather less firm than the texture of the mass. Not more than five ounces of blood was lost during the operation, which lasted over an hour, and was not attended with a great deal of pain—a dragging sensation at the hypogastrium being the chief suffering complained of, with some severe twinges in the region of the left kidney and spleen, during the latter part of the process.

After the removal of the tumor, the os tincæ, which remained largely dilated, but thin and flaccid like the mouth of a gum-elastic bag, could be seen from the vulva. I introduced a finger into its cavity (which was found rough and cellular to the touch), in order to carry the uterus up to its proper position. The patient was then drawn up in bed, directed to be kept perfectly quiet, and take a tablespoonful of neutral mixture with the tenth of a grain of morphia, every hour, till she should fall into perspiration and be composed to sleep. On the following day she was found free of pain, and comfortable. Some slight soreness was felt in the hypogastrium during the next, which was removed by a single warm fomentation. A slight oozing from the uterus took place during the four or five days succeeding the operation —no further inconvenience followed. The patient was. however, kept in bed for three weeks, in order to obviate any tendency to prolapsus which might otherwise have taken place. At that period, she resumed her usual occupations, and has remained, up to that time, as healthy and active as she has been at any period of her life.

In its character and mode of attachment to the uterus, the tumor removed in this case was somewhat UNIQUE. It was not surrounded by a mucous membrane or pediculated like an ordinary polypus. It contained no vesicles or fœtal remains like the *ordinary* moliform productions. It was solid, very friable and readily split in layers, appearing to be made up of a mass of gelatinous fibres interlaid with crude opaque albuminous matter. It was of a pale rose hue; this color being in all probability due to the presence of the small vessels which gave out blood during the extraction of the tumor, though no vessels were visible on making an inspection of the diseased mass after its removal.

Tumors of a character somewhat similar to this, have been supposed to arise from the organization of coagulated blood in the cavity of the uterus. But in this instance it seems to me more probable, that it has been gradually developed in the form of a morbid exudation from the cavity of the uterus, which exudation having received, to more or less extent, vessels from the uterus, participated in the menstrual engorgement of this organ, and allowed the menstrual fluid to escape.

*Philadelphia*, *Sept.* 24, 1844.

